# Editorial

**Published:** 1839-01-12

**Authors:** 


THE MEDICAL EXAMINER.

PHILADELPHIA, JAN. 12, 1839.

Dr. Broussais, the founder of the Physiological Doctrine of Medicine, died at Paris, on the 17th of November, of a cancerous disease of the rectum.

The labours of Broussais have left so deep an impression upon the science of medicine, that we cannot pass over his death in silence. Like all other founders of exclusive systems, he could scarcely be appreciated during his life. On the one hand his zealous admirers, and on the other his opponents, were disposed to exalt or to depreciate his medical reputation, according to the partial standard by which they were governed. The physiological doctrine of disease had already passed through its complete revolution before the death of Broussais. At first earnestly opposed, it soon became the system, which, in France, attracted the most zealous, if not the most numerous followers,—and, for a while, it seemed destined to become the predominant system of medicine. The physiological doctrine, during its most brilliant period, was not limited to France, nor, indeed, to Europe;—it seemed to possess a power of universal application, and was received with enthusiasm in many countries of the American continent, especially those in which the violent febrile diseases of tropical climates are most destructive.

During the rapid progress of the physiological doctrine, its author was earnestly and constantly engaged in an incessant polemical warfare with its opponents. For him, there was no medium; the physiological doctrine of medicine solved all difficulties, simplified therapeutics, and, with a few formulæ, enabled the young and inexperienced physician to combat disease with more success than the veteran practitioner, who had toiled through a long life of patient observation. The high talents, and energetic, impetuous character of Broussais, sustained him in this endless controversy, and placed him and his school in an attitude of hostility to established opinions, which soon gained for him numerous disciples. Of these, some adhered to his doctrine from love of its apparent simplicity,—others joined the physiological school, from that fondness for distinction which is most easily gratified by becoming a proselyte to the opinions which are most novel, and therefore attractive.

The onward progress of the physiological school continued as long as its followers were freely examining one after another the medical opinions which had been based upon the accumulated experience of centuries, and in substituting for them the new combinations of irritation or inflammation. But a single leading idea is soon exhausted,—and, however much the nomenclature of diseases might be varied by referring constantly to the organ which is chiefly affected, it became very evident that in all cases there was some modification of inflammatory action, which was to be removed by the same system of therapeutics. The medical profession soon began to tire of these continual repetitions, and very soon discovered that, with some trivial exceptions, all works on the physiological doctrine consisted only of an incessant reiteration of the same theme, with a few unimportant variations.

An epoch in the history of the physiological school occurred, when Broussais became Professor of the Faculty of Medicine of Paris. A new chair was founded for him at the revolution of July, that of General Pathology and Therapeutics. This appointment was a sort of acknowledgement of the standing of the physiological school, and, as it were, a public recognition of the system. There were never more than two or three professors of the School of Medicine of Paris who entirely adopted the opinions of Broussais; but his admission into the faculty, was, in itself, a signal triumph.

Much to the surprise of every one, the physiological system declined with increased rapidity from that moment;—the endless repetitions of the new professor wearied, while his fondness for sarcasm, and, at times, for gross personalities, disgusted his audience; his class became less and less numerous, and, for some years, was one of the smallest at the School of Medicine. About the same time that Broussais was appointed professor, the reaction against his system became more decided;—a number of observing men had been studying disease with the aid of pathological anatomy and the new means of investigation which Laennec had discovered; the results of their inquiries were published, and proved that Broussais had been carried vastly beyond the deductions which could fairly be drawn from observation. It soon became clear to those who were most attached to the physiological doctrine, that the nature of a disease was not always explained by calling it an irritation or an inflammation, and that leeches and demulcents could not replace the whole materia medica. As a system, the physiological doctrine can scarcely be said to exist; very few of its ultra adherents can now be found, except amongst the French army surgeons, and in some parts of the tropical region of America. That its existence as a system should cease, was natural enough; a similar fate has befallen others, which, for a time, have had equal notoriety with the doctrines of Broussais.

We must not, however, examine the opinions of Broussais as a system; however defective they may be as a whole, in many respects they have produed a most salutary reform in medicine. Had Broussais contented himself with the publication of his works on "Chronic Inflammation," and the "Examination of Medical Doctrines," the good which he has done, would have been unmixed with evil, and it is to these earlier works that we must look for the best evidence of the genius of the author. His later productions are much more feeble and less interesting.

We have already alluded to the indirect good which resulted from the works of Broussais. Many experienced and observing physicians could not adopt the opinions of Broussais, while they were unable to oppose them, except by pursuing a new course of observation on the diseases which he had treated of most largely. We are, therefore, in some measure indebted to Broussais, for the admirable works of Louis, Chomel, and Andral, which furnish the only satisfactory reply to his exaggerated notions. It would, however, be doing great injustice to his memory, to pass over the positive and direct good which has resulted from his writings. He has enforced the examination of the suffering organs in febrile diseases—has explained many of their sympathies—has shown that with perseverance in a mild antiphlogistic treatment many chronic affections will disappear—and, above all, has prevented the abuse of irritating remedies in inflammatory diseases. These results are the most important, and will be finally received amongst the admitted maxims of medicine, but they are not the only good which Broussais has done; a vast number of diseases have become better known through his labours, and the novel points of view from which he has examined them. He has rarely studied them with an unbiassed mind, but while we make the necessary allowance for his peculiar views, we can in every case gather a vast amount of practical knowledge, which is rendered the more impressive from the earnest, energetic language in which it is clothed.

